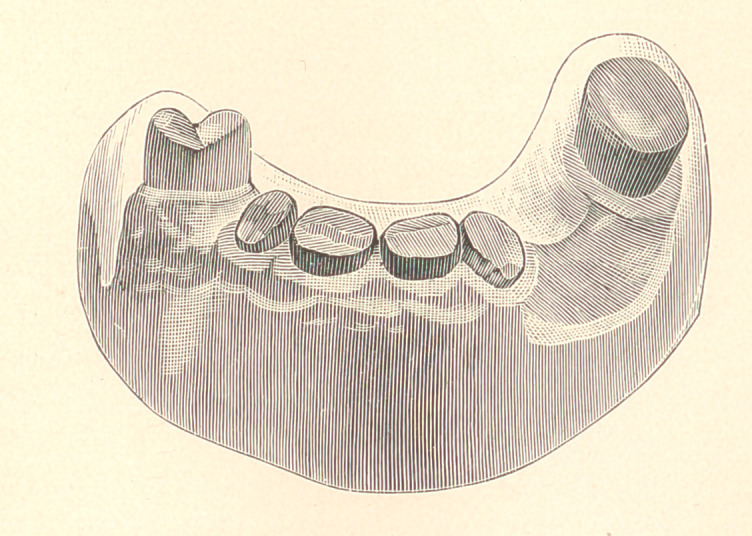# Crown- and Bridge-Work

**Published:** 1892-12

**Authors:** C. M. Richmond

**Affiliations:** New York


### CROWN- AND BRIDGE-WORK.[1]

BY DR. C. M. RICHMOND, NEW YORK.

(Continued from page 792.)

In the present article I have illustrated a novel operation. The four incisors are worn down very near to the gum-line, and all have the pulps alive. The surfaces were cut and smoothed as much as the patient would endure, as they were extremely sensitive, being of a peculiar temperament that could not tolerate a root with the pulp out. It was decided to restore the case with a movable bridge.

The molar and bicuspid were first crowned with gold or fitted with gold crowns, leaving them uncemented until the case was finished; then they were placed into position in the bridge, and were cemented on as if it were to be a fixed piece, the reason being that from the irregular position of the two teeth in relation to one another and to the bands, it would be impossible to secure the bearings right in any other way. The case then could be removed without trouble.

After the crowns were finished they were placed, cutting end down, into plaster, and after becoming sufficiently hard the sides were cut away, leaving a small, square block of plaster with the gold crowns embedded in it. The plaster was then cut slightly on each side and on the end, and then the block was broken apart and the crowns removed. The two broken pieces were tied together with silk and a perfect model of each cast with fusible metal. (When I refer to fusible metal, I mean that made by R. S. Williams from the formula I gave him, also published in one of my articles, as it is the only metal I know of that is hard enough to endure the work required, it being as hard as zinc.) The bands were now made on these dies in the way described for constructing the double band, and fitted perfectly, remaining closed and slipping on and off quite hard. An impression was taken of the incisor roots, and a die

---

[1] Copyrighted, 1892, by Dr. C. M. Richmond.

made in the same way as the others, all in one model, however. A [illegible] of pure gold is burnished on to this to the gum-line on the palatine and to the front edge of the facial surfaces. As the teeth were to be fitted to the roots, and the roots to make one-quarter of the exposed surfaces, very irregular curved teeth were resorted to, in order to secure the shades and shapes to suit the case. After six or seven trials a most perfect match and fit were obtained. The teeth to fill in the space posterior to the bicuspid and anterior to the molar were also carved in blocks, with the gum baked on to make the finishing surfaces perfect. The five front teeth were fitted and soldered to the gold cover made for the front teeth; the curved blocks were fitted and soldered to gold plates made to fit the gum surfaces where the spaces were to be filled, in the way described, by pure gold being burnished on to dies.

The case was now put into the mouth in sections, the molar block waxed to the bicuspid band, the crown placed on in its proper position, and the part then tried on. The teeth were closed and the block arranged in its proper place. It was then carefully removed and invested, and the block soldered to the band. The block anterior to the molar was tried in the same way, and when in its proper position was also carefully removed, invested, and this block soldered to the molar band. Each block was so arranged that we could wax the whole case together and try in one piece. In doing this the front piece was waxed to one side first, and inserted to have it properly adjusted. Then, after removing, a piece of wire was heated and dropped into the wax, melting its way into it, and in this way the case was held together strong enough to try on and adjust, without its breaking apart at the joint where it was to be soldered. This was placed into an investment and soldered, and the last trial was made of the pieces in the mouth. The pieces were waxed together; the two gold crowns were placed into position in the work and tried in the mouth. This brought the teeth into position, and the whole case was in readiness for the last impression, which was taken as a matrix guide, to be used after it was ready for the last investment. After taking the case off, it was placed in the investment and soldered for the last time.

It will be seen, in referring to the plaster model, that the case looks impossible from the situation of the teeth, the molar standing out of the arch, and also having a forward pitch. The bicuspid is almost in its natural position, yet the case would go on and off by starting the molar-band first, and when in position was held absolutely firm.

The patient visited my office three times and saw the methods employed in constructing such cases, and, being a dentist, returned to his office, and by the aid of an assistant constructed the entire case himself. The only part I did was to finish the operation by cementing the gold crowns, as before described.

Dr. C. P. Wilson, 85 Newberry Street, Boston, is the dentist referred to, and any one who wishes to see one of the finest pieces of dental work ever constructed should visit him and request him to exhibit it. The front teeth are so well fitted that no one can detect where the joint is without a close examination.

I have had some new spatulas made for mixing cement. I found that the acid would at once attack steel, and as soon as the nickel was worn off a chemical action would at once take place. I now use copper or nickel for spatulas for all cements. I also use a cube of glass instead of a slab. A three-inch cube can be placed in hot water a moment, and sufficient heat will be absorbed, so that all trouble from thermal changes is at once avoided.

(To be continued.)

## Figures and Tables

**Figure f1:**
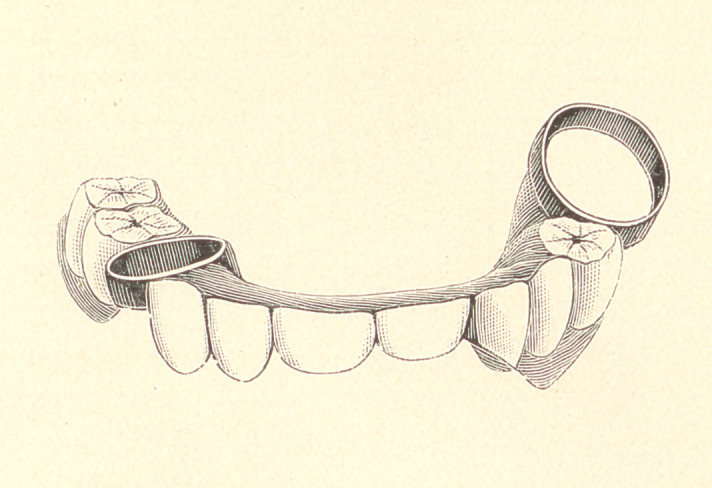


**Figure f2:**